# Diet and Functional Gastrointestinal Disorders in Children. Is the Focus on Food Exaggerated?

**DOI:** 10.3390/nu11020250

**Published:** 2019-01-23

**Authors:** Amanda C. Fifi, Miguel Saps

**Affiliations:** Division of Pediatric Gastroenterology, Hepatology & Nutrition, Department of Pediatrics, Miller School of Medicine, University of Miami, Miami, FL 33136, USA; msaps@med.miami.edu

Almost all children (93%) who consult for irritable bowel syndrome (IBS) report food intolerances. In a large number of cases, there is poor correlation between the self-perceived intolerances and the frequency or severity of pain [1]. The self-perceived limitations lead to unnecessary restrictions on food types. Interestingly, self-perceived intolerance to foods is also common among healthy children, with almost two in three healthy children perceiving intolerances and limiting their diet [1].

Among the most commonly reported intolerances in children with IBS are dairy products. Double blind placebo controlled trials showed no relation between lactose intolerance and recurrent abdominal pain in children [2]. Lately, adults and children report intolerance to gluten. Studies from Argentina [3] and the Netherlands [4] have found that almost one in ten individuals report gluten intolerance, with a large proportion of them restricting gluten without medical indication. This has enlarged the market for “gluten free” products and the perception by the public that gluten is detrimental. With the exception of approximately 1% of the population that have celiac disease [5], exclusion of gluten is rarely indicated. A study from Australia showed that if adult patients first excluded fermentable, oligosaccharides, disaccharides, monosaccharides, and polyols (FODMAPs), the additional exclusion of gluten does not further reduce symptoms [6]. This suggests that since gluten is frequently found in the FODMAPs group of foods, intolerance to FODMAPS may be confused with intolerance to gluten.

Studies in children have shown that FODMAPs may trigger symptoms in patients with IBS [7]. This is believed to be, at least in part, related to colonic gas production and subsequent distension that results in discomfort and abdominal pain in patients with visceral hypersensitivity [8]. Patients with visceral hypersensitivity may be also more prone to develop discomfort and pain in the setting of other intolerances to disaccharides. This hypersensitivity can explain the occurrence of abdominal pain in patients with lactose intolerance, even though lactose intolerance itself is not the direct cause of functional abdominal pain in children. Similarly, the presence of gastrointestinal symptoms in children with genetic sucrose-isomaltase deficiency has been reported [9]. Both are examples of how association, even if true, does not necessarily mean causation. The belief that some food groups cause harm has been perpetuated by conventional and social media. Self-proclaimed “health gurus” frequently recommend excluding different types of foods based on anecdotal evidence and personal experience. It can be dangerous to advise children to unnecessarily eliminate certain foods for prolonged periods, because this places them at risk for nutritional imbalances including fiber, vitamin, and mineral deficiencies. Moreover, though still incompletely understood, modifications in diet may result in changes in the microbiome [10]. 

The problem of restrictive diets is not limited to those who eliminate without an indication. Even in those in whom transient restriction of certain food groups may be justified, an additional and not yet answered question is, when and how to reintroduce foods. In the case of FODMAPs, although a common recommendation is to reintroduce FODMAPs progressively and to continue to eliminate the foods that trigger symptoms [11], the instrumentation of this plan may not be easy, as patients do not easily recognize the offending food. The lack of efficacy of current conventional treatments for functional abdominal pain disorders (FAPDs) leads some patients and their families to seek out complementary alternative medicine, specifically the use of herbs and spices for relief. Though herbs and spices have historically been believed to play a role in treatment of many gastrointestinal and pain disorders, their use as FAPDs, especially in pediatrics, remains dubious and further randomized placebo controlled studies are needed to assess their effectiveness.

Myths and misconceptions are not only limited to children with abdominal pain. The recommendation for increased water and fiber as part of the treatment of functional constipation in children is not based on sufficient evidence. Most water is reabsorbed before reaching the rectum, thus the utility of increasing water to soften the stools is questionable. Additionally, systematic reviews conducted in children found little evidence for recommending fiber [12]. Furthermore, the latest guidelines from the European Society of Pediatric Gastroenterology, Hepatology and Nutrition (ESPGHAN)/North American Society of Pediatric Gastroenterology, Hepatology and Nutrition (NASPGHAN) do not recommend increasing fiber in excess of the normal daily requirement for the child’s weight and age [13]. Despite the lack of sufficient evidence for water and fiber supplementation in children with constipation, 86% of pediatric gastroenterologist recommend to increase water, and 81% of them to increase fiber for the treatment of functional constipation [14].

In order to provide updated evidence, in the midst of confusion, for the benefit of dietary interventions in functional gastrointestinal disorders in children, we have invited an international panel of pediatric gastroenterologists and dieticians to review the literature and to provide their opinion on the benefit of dietary measures for their treatment. We hope that this issue will clarify some of the myths and misconceptions, to provide the practitioner with valuable information and the researcher with a list of yet unanswered questions that could direct future investigations.
